# Endometrial thickness is associated with low birthweight in frozen embryo transfer cycles: A retrospective cohort study of 8,235 singleton newborns

**DOI:** 10.3389/fendo.2022.929617

**Published:** 2022-10-28

**Authors:** Tingting He, Mingzhao Li, Wei Li, Peng Meng, Xia Xue, Juanzi Shi

**Affiliations:** Assisted Reproduction Center, Northwest Women’s and Children’s Hospital, Xi’an, China

**Keywords:** Endometrial thickness, low birthweight, frozen embryo transfer, singleton, newborns

## Abstract

**Objective:**

To explore the association between endometrial thickness (EMT) and adverse neonatal outcomes in frozen *in vitro* fertilization/intracytoplasmic sperm injection-embryo transfer (IVF/ICSI-ET) cycles.

**Methods:**

This retrospective study involved a total of 8,235 women under the age of 35 years who underwent IVF/ICSI cycles and received frozen embryo transfer (FET) at a tertiary-care academic medical from January 2015 to December 2019, resulting in a live singleton newborn. Patients were categorized into three groups depending on EMT: ≤7.5 mm, 7.5-12 mm and >12 mm. The primary outcome was low birthweight (LBW). The secondary outcomes were preterm birth (PTB), small-for-gestational age (SGA), large-for-gestational age (LGA) and high birthweight (HBW).

**Result(s):**

Compared with EMT >7.5–12 mm group, the risk of being born LBW was statistically significantly increased in the EMT ≤7.5 mm group (adjusted odds ratio [aOR] 2.179; 95% confidence interval [CI], 1.305–3.640; P=.003), while dramatically decreased in the EMT >12 mm group (aOR 0.584; 95% CI, 0.403-0.844; P=.004). Moreover, newborn gender and pregnancy complications were all independent predictors for LBW. Furthermore, a significant decrease in birthweight was found in the EMT ≤7.5 mm group as compared with EMT >7.5–12 mm group and EMT >12 mm group (3,239 ± 612 vs. 3,357 ± 512 and 3,374 ± 479 g, respectively), and similar result was found in term of gestational age (38.41 ± 2.19 vs. 39.01 ± 1.68 and 39.09 ± 1.5 weeks, respectively).

**Conclusion(s):**

After frozen IVF/ICSI-ET, EMT ≤7.5 mm is independently associated with increased risk of LBW among women with singleton newborns. Therefore, we suggest that women with EMT ≤7.5 mm after achieving pregnancy by IVF/ICSI-ET treatment should warrant more attention to reduce the risk of delivering a LBW newborn.

## Introduction

With the worldwide use of assisted reproductive technology (ART), concerns about the health of these children have prompted various groups to study the perinatal risks of pregnancy. It has been reported that infants after ART conception have an increased risk of adverse neonatal outcomes, such as low birthweight (LBW), preterm birth (PTB) as well as small-for-gestational age (SGA), when compared with those spontaneously conceived, even for singleton births (1, 2). However, the biological mechanism of adverse neonatal outcomes is not entirely understood. On the one hand, some studies have shown that subfertility itself is the main reason for the adverse perinatal outcomes (3, 4). On the other hand, growing evidence suggested that both ART procedures and embryo manipulation in the laboratory may play a vital role (5–8). Furthermore, recent studies reported that endometrial thickness (EMT) could be involved as well.

EMT is routinely measured by transvaginal ultrasound (TVU) during infertility treatment to assess uterine receptivity. Thin EMT is common but challenging occurrence in assisted reproduction, which was mainly caused by Asherman syndrome, history of uterine surgery, infection or radiation (9). As far as we know, most of the previous studies focused on the relationship between pregnancy outcomes and EMT, and found that women with thin EMT had a lower clinical pregnancy and live birth rates in both fresh and frozen embryo transfer (FET) (10, 11). Only limited studies explored the effects of EMT on adverse neonatal outcomes, and their findings were inconsistent. Guo et al. and Zhang et al. demonstrated that EMT was an independent predictor for SGA and LBW, but Oron et al. did not find any association which were mainly caused by sample size, fresh or frozen cycles, the classification of endometrial thickness and whether exclusion women with pregnancy complications (12–14).

It has been hypothesized that FET may provide a more favorable intrauterine environment for embryo implantation and placentation by avoiding the supraphysiologic condition that occurred after ovarian stimulation, which has been considered as an independent predictor of LBW in fresh cycles (15, 16). With the introduction of more efficient cryopreservation techniques, an increasing number of studies has shown better results after FET than after fresh cycles (17–19). As a consequence, the rate of FET has steadily been rising worldwide.

However, the previous studies regarding neonatal outcomes mainly focused on fresh cycles. In addition, it prevented them from drawing solid conclusion without excluding women with hypertension, which contribute to adverse pregnancy outcome (20). Therefore, the aim of the present study was to comprehensively evaluate the association between EMT and neonatal outcomes of in women undergoing FET cycles.

## Methods

### Study design and participants

This was a retrospective cohort study conducted at the Center for Assisted Reproductive Technology of Northwest Women’s and Children’s Hospital, People’s Republic of China, from January 2015 to December 2019. Patients were considered eligible if they met the following criteria: [1] <35 years of age; [2] undergoing FET cycles and having alive singleton birth ≥28 weeks. Exclusion criteria were [1] multiple pregnancies, vanishing twins and still birth; [2] Cycles with oocyte donation; [3] preimplantation genetic testing cycles; [4] congenital uterine malformations or acquired uterine diseases (untreated endometrial polyps, submucosal fibroids, intrauterine adhesions); [5] women with chronic hypertension. In addition, if more than one delivery for the same women was in the electronic database, only the first pregnancy was included for analysis. This study was approved by the Ethics Committee of the Northwest Women’s and Children’s Hospital (number 2022007) and formal written consent was obtained from each patient.

### Laboratory and IVF/ICSI protocols

The protocol for ovarian stimulation was determined based on the patient’s age, body mass index (BMI), antral follicle count (AFC), and basal follicle stimulating hormone. Most patients were treated with GnRH antagonist or prolonged or long gonadotropin-releasing hormone (GnRH) agonist protocol, while women with diminished ovarian reserve, the mild ovulation protocol was attempted. When more than two follicles reached a mean diameter of 17 mm, human chorionic gonadotropin (hCG) was administrated to induce oocyte maturation at a dose of 5,000 to 10,000 IU. Oocytes retrieval was performed 36 h later, followed by conventional IVF or ICSI based on the male partner’s semen quality. Details on IVF/ICSI procedures, embryo culture and the embryo scoring system have been previously described (21). The verification, warming procedure, and embryos transfer procedures was performed according to standard protocols (22). Luteal phase support was started after oocyte retrieval and was continued until 10 weeks’ gestation.

### Endometrial preparation and thickness assessment

The type of endometrial preparation was determined according to the experience of the physician, based on patients’ characteristics, including natural cycle (NC), hormone replacement therapy (HRT) and GnRH agonist combined with HRT (GnRH agonist-HRT) protocol. In short, women with regular menstrual cycles were allocated to NC, while patients having irregular cycles were offered either HRT or GnRH agonist-HRT protocol. The detailed protocols for endometrial preparation were described in our previous studies (23). To guarantee the accuracy and reliability as possible, EMT was measured by highly trained and experienced sonographers of the same team *via* Voluson E8 (GE Healthcare, Australia) with intracavity probes. We identified EMT by the largest diameter from one endometrial–myometrial interface to the other in the midsagittal plane. In NC cycles, EMT was measured on the day of hCG administration, while in women with HRT or GnRH agonist-HRT protocol, EMT was recorded from the last ultrasound prior progesterone initiation. Patients were categorized into three groups according to EMT: ≤7.5 mm, 7.5-12 mm and >12 mm, and 7.5-12 mm served as a reference group. These thresholds were selected based on the previous studies (12, 14, 24).

### Outcome measures

Live birth was defined as the delivery of a viable infant ≥ 28 weeks of gestational age. In our study, the primary outcome was LBW, and the second outcomes included very preterm birth (VPTB), PTB, very small-for-gestational age (VSGA), SGA, very large-for-gestational age (VLGA), LGA, very low birthweight (VLBW) and high birthweight (HBW). VPTB and PTB were defined as delivery before 32 and 37 completed gestational weeks, respectively. We categorized birthweight as normal (≥2500g and ≤4000g), VLBW (<1500g), LBW (<2500g) and HBW (>4000g). The outcomes of VSGA, SGA, LGA, and VLGA were respectively defined according to birthweight for the 3rd, 10th, 90th and 97th percentile of gender-specific birthweight reference for Chinese (25). We also assessed pregnancy complications among singleton live births, including gestational diabetes mellitus (GDM), hypertensive disorders, placenta previa and placental abruption. Data on pregnancy and neonatal outcomes were obtained from electronic medical records and the follow-up system in our center has been described in detail previously (26).

### Statistical analysis

All statistical analyses were performed with IBM SPSS Statistics version 25. Normally distributed continuous variables were expressed as mean and standard deviation (SD) and the comparison of the study groups was performed with one way analysis of variance (ANOVA). Continuous variables with non-normally distributions were represented as medians and interquartile ranges (IQR), and the differences between study groups were calculated using Kruskal-Wallis test. Categorical data were shown in frequencies and percentages, and differences between study groups were compared using Chi-squared test or Fisher’s exact test, as appropriate. *P*<0.05 was considered statistically significant. *Post hoc* pairwise comparison was performed by Bonferroni’s correction.

Univariate and multivariate regression analyses were used to assess the effects of EMT on the risk of LBW and other neonatal outcomes. The potential confounders were chosen based on clinical experience and studies published in recent years, including maternal age, maternal BMI, infertility duration, parity, FET cycle rank, infertility type, infertility cause, EMT, fertilization type, endometrial preparation protocols, stage of embryo transferred, number of transferred embryos, number of good-quality embryo transfer, cesarean delivery, newborn gender and pregnancy complications, such as hypertensive disorders and GDM. EMT >7.5–12 mm was chosen as a reference group. The results were presented as odds ratio (OR), adjusted odds ratio (aOR), and confidence interval (CI).

## Results

### Participant characteristics

**Figure 1:**
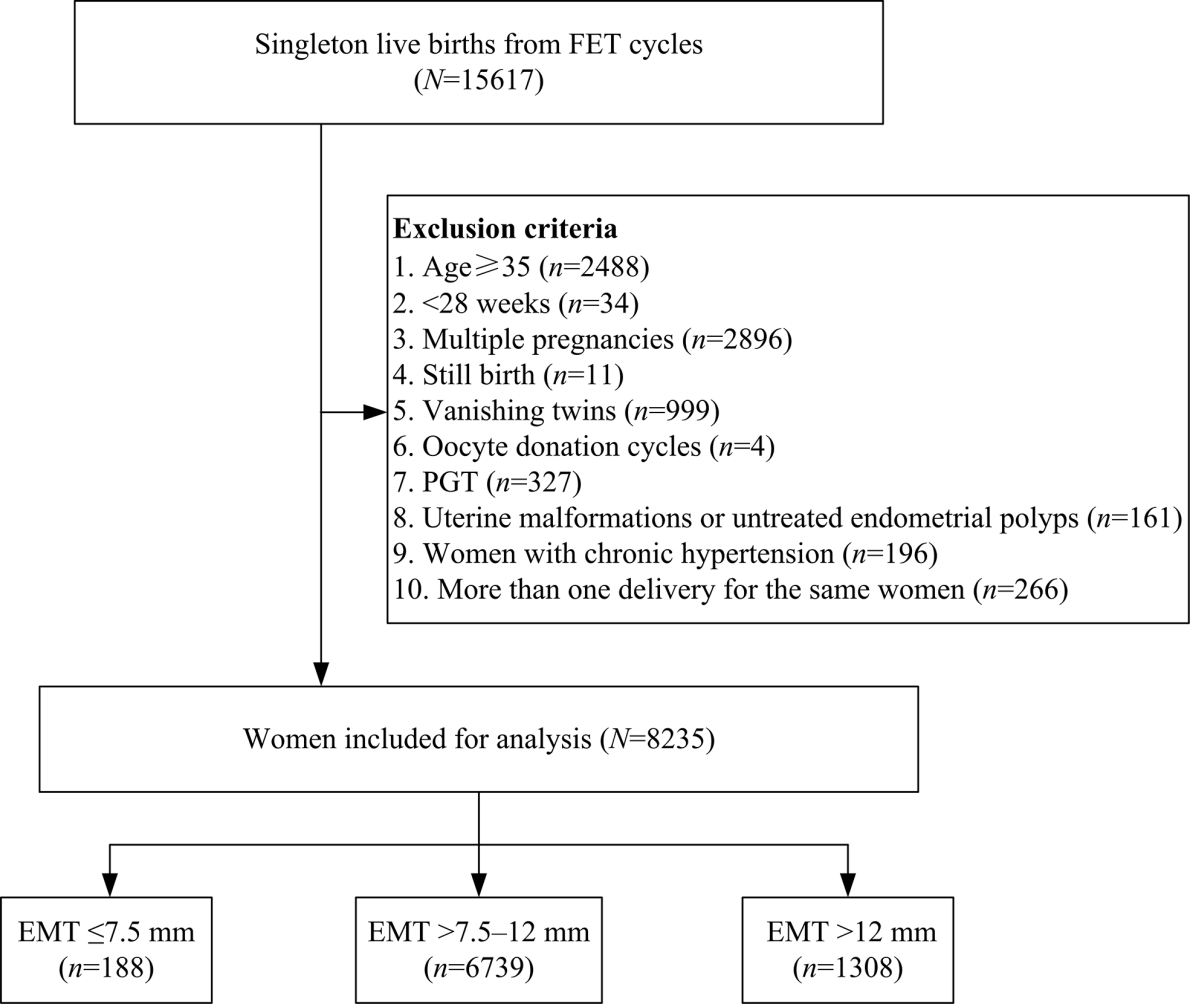
Flow chart of the study.

A total number of 15,617 singleton live births resulting from FET cycles were screened from our database, and 7,382 cycles were excluded as detailed in **Figure 1**. Of the remaining 8,235 cycles, EMT ≤7.5 mm was observed in 188 (2.3%) women, while the number of patients with EMT >7.5–12 mm and EMT >12 mm was 6,739 (81.8%) and 1,308 (15.9%), respectively. Baseline characteristics according to EMT stratification were presented in **Table 1**. Maternal age, maternal BMI, infertility duration, FET cycle rank, infertility type, infertility cause, fertilization type, endometrial preparation protocols, number of transferred embryos as well as pregnancy complications were statistically significantly different between the groups. However, there were no significant differences in parity, stage of embryo transferred as well as number of good-quality embryo transfer among the groups.

**Table 1:** Baseline characteristics according to EMT stratification.

Characteristics	EMT ≤ 7.5	7.5<EMT ≤ 12	EMT>12	*P* value
	(*n*=188)	(*n*=6,739)	(*n*=1,308)	
Maternal age (y)	29.77 ± 2.69	29.33 ± 2.82	29.49 ± 2.81	0.021^*^
Maternal BMI (kg/m2)	21.99 ± 3.14	22.15 ± 3.2	22.56 ± 3.31	<0.001^*,^ ^c^
Infertility duration (y)	2.49 (1.46,3.84)	2.91 (1.94,4.37)	3.02 (1.91,4.36)	0.001^*,^ ^a^ ^,^ ^b^
Parity, n (%)				0.41
0	169 (89.9)	6,216 (92.2)	1,199 (91.7)	
≥1	19 (10.1)	523 (7.8)	109 (8.3)	
FET cycle rank, n (%)				0.001^*,^ ^a^ ^,^ ^b^
0	126 (67)	5,231 (77.6)	1,032 (78.9)	
≥1	62 (33)	1,508 (22.4)	276 (21.1)	
Infertility type, n (%)				<0.001^*,^ ^a^ ^,^ ^b^ ^,^ ^c^
Primary	78 (41.5)	4,103 (60.9)	893 (68.3)	
Secondary	110 (58.5)	2,636 (39.1)	415 (31.7)	
Infertility cause, n (%)				<0.001^*^
Female factor	143 (76.1)	4,266 (63.3)	790 (60.4)	
Male factor	14 (7.4)	1,255 (18.6)	267 (20.4)	
Mixed	22 (11.7)	929 (13.8)	201 (15.4)	
Unexplained	9 (4.8)	289 (4.3)	50 (3.8)	
Fertilization type, n (%)				0.01^*,^ ^a^ ^,^ ^b^
IVF	160 (85.1)	5,094 (75.6)	986 (75.4)	
ICSI	28 (14.9)	1,645 (24.4)	322 (24.6)	
Endometrial preparation protocols, n (%)				<0.001^*,^ ^b^ ^,^ ^c^
NC	18 (9.6)	824 (12.2)	416 (31.8)	
HRT	117 (62.2)	4,033 (59.8)	481 (36.8)	
GnRH agonist-HRT	53 (28.2)	1,882 (27.9)	411 (31.4)	
No. of transferred embryos, n (%)				<0.001^*,^ ^c^
1	103 (54.8)	3,640 (54)	810 (61.9)	
2	85 (45.2)	3,099 (46)	498 (38.1)	
Stage of embryo transferred, n (%)				0.21
Cleavage stage	45 (23.9)	1,976 (29.3)	368 (28.1)	
Blastocyst stage	143 (76.1)	4,763 (70.7)	940 (71.9)	
No. of good-quality embryo transfer, n (%)				0.19
0	107 (56.9)	3,417 (50.7)	651 (49.8)	
≥1	81 (43.1)	3,322 (49.3)	657 (50.2)	
Pregnancy complication, n (%)	35 (18.6)	668 (9.9)	139 (10.6)	<0.001^*,^ ^a^ ^,^ ^b^

Values are presented as mean ± standard deviation or medians (interquartile ranges) or number (percentage).

BMI, body mass index; FET, frozen embryo transfer; IVF, in vitro fertilization; ICSI, intracytoplasmic sperm injection; NC, natural cycle; HRT, hormone replacement therapy; GnRH gonadotropin-releasing hormone.

^*^P < 0.05 was considered statistically significant.

a Statistically significant differences between EMT ≤7.5 mm group and EMT >7.5–12 mm group.

b Statistically significant differences between EMT ≤7.5 mm group and EMT >12 mm group.

c Statistically significant differences between EMT >7.5–12 mm and EMT >12 mm group.

### Neonatal outcomes

The neonatal outcomes for singleton live births based on EMT were shown in **Table 2**. The mean gestational age (38.41 ± 2.19 vs. 39.01 ± 1.68 and 39.09 ± 1.5 weeks, respectively; P<0.05) and birthweight (3,239 ± 612 vs. 3,357 ± 512 and 3,374 ± 479 g, respectively; P<0.05) were significantly decreased in EMT ≤7.5 mm group as compared with EMT >7.5–12 mm group and EMT >12 mm group. The incidence of VPTB in the EMT ≤7.5 mm group (2.7%) was significantly higher than that in the EMT >7.5–12 mm group (0.8%) and the EMT >12 mm group (0.4%), and similar result was found for PTB (18.6% vs. 8%; 18.6 vs. 7%, respectively, P<0.05). However, no significant differences were found between the EMT >7.5–12 mm and EMT >12 mm groups in the terms of gestational age, birthweight, VPTB as well as PTB. The incidence of VLBW in women with EMT ≤ 7.5mm was significantly higher than that in women with EMT>12 mm (2.1% vs. 0.2%, P<0.05). The incidence of LBW was significantly decreased with the increase of EMT (10.6% vs 4.4% vs 2.7%, respectively; P<0.05). At the same time, we noticed that cesarean delivery varied significantly according to EMT. However, there was no significant difference in the incidence of female gender, VSGA, SGA, LGA, VLGA and HBW.

**Table 2:** Neonatal outcomes for singleton live births based on endometrial thickness.

Neonatal outcomes	EMT ≤ 7.5	7.5<EMT ≤ 12	EMT>12	*P* value
	(*n*=188)	(*n*=6,739)	(*n*=1,308)	
Gestational age (week)	38.41 ± 2.19	39.01 ± 1.68	39.09 ± 1.5	<0.001^*,^ ^a^ ^,^ ^b^
Birth weight (g)	3,239 ± 612	3,357 ± 512	3,374 ± 479	0.008^*,^ ^a^ ^,^ ^b^
Cesarean delivery, n (%)	153 (81.4)	5,070 (75.2)	947 (72.4)	0.011^*^
Male gender, n (%)	108 (57.4)	3,647 (54.1)	696 (53.2)	0.53
Very preterm birth, n (%)	5 (2.7)	55 (0.8)	5 (0.4)	0.004^*,^ ^a^ ^,^ ^b^
Preterm birth, (%)	35 (18.6)	540 (8)	91 (7)	<0.001^*,^ ^a^ ^,^ ^b^
Very small for gestational age, n (%)	1 (0.5)	110 (1.6)	18 (1.4)	0.5
Small for gestational age, n (%)	11 (5.9)	331 (4.9)	61 (4.7)	0.77
Large for gestational age, n (%)	27 (14.4)	1,155 (17.1)	244 (18.7)	0.23
Very Large for gestational age, n (%)	10 (5.3)	441 (6.5)	104 (8)	0.13
Very low birth weight (<1,500 g), n (%)	4 (2.1)	43 (0.6)	2 (0.2)	0.003^*,^ ^b^
Low birth weight (<2,500 g), n (%)	20 (10.6)	299 (4.4)	35 (2.7)	<0.001^*,^ ^a^ ^,^ ^b^ ^,^ ^c^
High birth weight (>4,000 g), n (%)	9 (4.8)	491 (7.3)	102 (7.8)	0.33

Values are presented as mean ± standard deviation or number (percentage).

^*^P < 0.05 was considered statistically significant.

a Statistically significant differences between EMT ≤7.5 mm group and EMT >7.5–12 mm group.

b Statistically significant differences between EMT ≤7.5 mm group and EMT >12 mm group.

c Statistically significant differences between EMT >7.5–12 mm and EMT >12 mm group.

To further investigate the relationship between EMT and LBW, univariate and multivariate regression analyses were performed. As demonstrated in **Table 3**, the incidence of LBW was statistically significantly increased in the EMT ≤7.5 mm group compared with those from the EMT <7.5-12 mm group (OR2.564; 95% CI, 1.59–4.135; P<.001), while dramatically decreased in EMT >12 mm group (OR0.592; 95% CI, 0.415–0.845; P=.004). BMI (OR1.035; 95% CI, 1.003–1.068; P=.034) and cesarean delivery (OR1.302; 95%CI, 1.002-1.693; P=.049) were positive predictors of LBW. In addition, newborn gender (OR1.467; 95%CI, 1.184-1.817; P<.001) and pregnancy complications (OR7.151; 95% CI, 5.706–8.961; P<.001) also significantly increased the risk of LBW.

**Table 3:** Odds ratios of LBW by univariate analysis of predictor variables.

Predictor variable	OR (95% CI)	*P* value
Maternal age	1.024(0.986-1.064)	0.226
Maternal BMI	1.035(1.003-1.068)	0.034^*^
Infertility duration	0.996(0.946-1.049)	0.883
Parity		
0	1	
≥1	0.688(0.435-1.088)	0.11
FET cycle rank		
0	1	
≥1	0.96(0.742-1.243)	0.759
Infertility type		
Primary	1	
Secondary	0.838(0.67-1.048)	0.121
Infertility cause		
Female factor	1	
Male factor	1.086(0.824-1.43)	0.558
Mixed	0.903(0.649-1.256)	0.545
Unexplained	1.46(0.921-2.317)	0.108
EMT		
7.5<EMT ≤ 12	1	
EMT ≤ 7.5	2.564(1.59-4.135)	<0.001^*^
EMT >12	0.592(0.415-0.845)	0.004^*^
Fertilization type		
IVF	1	
ICSI	1.036(0.81-1.326)	0.776
Endometrial preparation protocols		
NC	1	
HRT	1.329(0.951-1.856)	0.096
GnRH agonist-HRT	1.298(0.903-1.865)	0.159
No. of transferred embryos		
1	1	
2	0.962(0.776-1.192)	0.72
Stage of embryo transferred		
Cleavage stage	1	
Blastocyst stage	1.208(0.946-1.542)	0.129
No. of good-quality embryo transfer		
0	1	
≥1	1.068(0.863-1.321)	0.548
Cesarean delivery		
No	1	
Yes	1.302(1.002-1.693)	0.049^*^
Newborn gender		
Male	1	
Female	1.467(1.184-1.817)	<0.001^*^
Pregnancy complications		
No	1	
Yes	7.151(5.706-8.961)	<0.001^*^

OR, odds ratio; CI, confidence interval; BMI, body mass index; FET, frozen embryo transfer; EMT, endometrial thickness; IVF, in vitro fertilization; ICSI, intracytoplasmic sperm injection; NC, natural cycle; HRT, hormone replacement therapy; GnRH, gonadotropin-releasing hormone.

^*^P < 0.05 was considered statistically significant.

After adjusting for maternal age, maternal BMI, infertility duration, parity, FET cycle rank, infertility type, infertility cause, fertilization type, endometrial preparation protocols, stage of embryo transferred, number of transferred embryos, number of good-quality embryo transfer, cesarean delivery, newborn gender and pregnancy complications, EMT was still statistically significantly associated with LBW. As shown in **Table 4**, compared with EMT >7.5–12 mm group, the risk of being born LBW was statistically significantly increased in the EMT ≤ 7.5 mm group (aOR 2.179; 95% CI, 1.305–3.640; P=.003), while dramatically decreased in the EMT >12 mm group (aOR 0.584; 95% CI, 0.403-0.844; P=.004). Moreover, newborn gender (P=.001) and pregnancy complications (P<.001) were all independent predictors for LBW.

**Table 4:** Adjusted odds ratios of LBW by multivariate analysis of predictor variables.

Predictor variable	aOR (95% CI)	*P* value
Maternal age	1.02 (0.978-1.064)	0.362
EMT		
7.5<EMT ≤ 12	1	
EMT ≤ 7.5	2.179 (1.305-3.640)	0.003^*^
EMT >12	0.584 (0.403-0.844)	0.004^*^
Maternal BMI	1.004 (0.971-1.039)	0.799
Infertility duration	0.969 (0.916-1.026)	0.284
Parity		
0	1	
≥1	0.776 (0.469-1.286)	0.325
FET cycle rank		
0	1	
≥1	0.866 (0.654-1.147)	0.315
Infertility type		
Primary	1	
Secondary	0.801 (0.617-1.038)	0.093
Infertility cause		
Female factor	1	
Male factor	1.189 (0.852-1.659)	0.309
Mixed	0.952 (0.667-1.358)	0.785
Unexplained	1.311 (0.809-2.123)	0.272
Fertilization type		
IVF	1	
ICSI	0.956 (0.708-1.29)	0.767
Endometrial preparation protocols		
NC	1	
HRT	1.152 (0.811-1.636)	0.431
GnRH agonist-HRT	1.084 (0.74-1.589)	0.678
No. of transferred embryos		
1	1	
2	1.149 (0.897-1.471)	0.271
Stage of embryo transferred		
Cleavage stage	1	
Blastocyst stage	1.254 (0.953-1.65)	0.106
No. of good-quality embryo transfer		
0	1	
≥1	1.087 (0.865-1.367)	0.475
Cesarean delivery		
No	1	
Yes	1.082 (0.824-1.421)	0.570
Newborn gender		
Male	l	
Female	1.474 (1.182-1.838)	0.001^*^
Pregnancy complications		
No	1	
Yes	7.075 (5.591-8.953)	<0.001^*^

Adjustment included maternal age, maternal BMI, infertility duration, parity, FET, cycle rank, infertility type, infertility cause, fertilization type, endometrial preparation protocols, number of transferred embryos, stage of embryo transferred, number of good-quality embryo transfer, cesarean delivery, newborn gender and pregnancy complications. aOR, adjust odds ratio; CI, confidence interval; BMI, body mass index; FET, frozen embryo transfer; EMT, endometrial thickness; IVF, in vitro fertilization; ICSI, intracytoplasmic sperm injection; NC, natural cycle; HRT, hormone replacement therapy; GnRH, gonadotropin-releasing hormone.

^*^P < 0.05 was considered statistically significant.

## Discussion

In this retrospective cohort study of 8,235 singleton live births, we found that EMT was an independent risk factor for LBW. Furthermore, the birthweight and gestational age of neonates in EMT ≤ 7.5 mm group were significantly lower than those in EMT > 7.5-12 mm group and EMT >12 mm group. The relationship between EMT and neonatal outcomes has been investigated in several previous studies. The first study by Chung et al. found a twofold increased risk of LBW in EMT ≤10 mm group as compared with EMT >12 mm group (27). However, when the analysis was restricted to singleton live births, there was not significantly different. Du et al. demonstrated that EMT ≤7.5 mm was associated with an increased risk of LBW based on 2,847 singletons resulting from fresh cycles (24). However, it has been reported that supraphysiological E2 levels during ovarian stimulation created a suboptimal peri-implantation environment for implantation and placentation, thus leading to adverse perinatal outcomes such as LBW (16). A large retrospective study including 5,220 singleton newborns demonstrated that individuals with EMT less than 8 mm had an aOR of 1.57 (95% CI 1.09-2.26) for LBW in FET cycles (28). Nevertheless, this study was flawed by without ruling out of women with pregnancy complications, which are associated with a wide range of adverse neonatal outcomes even after adjusting a series of confounding factors in obstetric history and maternal characteristics (20).

This study, aiming to improve on the flaws of the abovementioned studies, explored the exact relationship between EMT and neonatal outcomes. Basing on 8,235 singleton newborns resulting from FET cycles, we found that the mean birthweight and gestational age were significantly decreased in the EMT ≤ 7.5 mm group as compared with groups with EMT >7.5–12 mm and EMT >12 mm. Furthermore, multivariate regression analysis showed that EMT, newborn gender and pregnancy complications were all independent risk factors for LBW.

The underlying mechanism of the impact of EMT on neonatal outcomes is still unclear and complex. It has been speculated that oxygen concentrations may play a role. Low oxygen tension in the intervillous space is crucial for normal embryo implantation and fetal development in the early pregnancy (29). Previous studies have demonstrated that the uterine spiral arteries contract after ovulation and lead to decreased blood flow to the surface of the endometrium, therefore reducing the oxygen concentration of functional epithelium during embryo implantation (24, 30). However, a thinned or absent functional layer may expose the embryos to higher vascularity and oxygen concentrations from the basal endometrium, thus pose a detrimental impact on embryonic and fetal growth.

Another mechanism is due to a defective vascular remodeling of the spiral arteries. These vessels have a unique importance because failure of their physiological transformation is considered to be a vital factor for reduced perfusion to the intervillous space and eventually resulting in pregnancy complications, such as preeclampsia and fetal growth restriction (31). Miwa et al. reported that a thin endometrium was characterized by high blood flow impedance of uterine radial artery, poor epithelial growth and poor vascular development (32). Therefore, we hypothesize that these adverse changes may affect the remodeling of uterine spiral artery, which in turn influence the development of the fetus and placenta, leading to the LBW outcomes observed in these women.

In this study, multivariate regression analysis showed that pregnancy complications and newborn gender were all independent predictors for LBW, which was consistent with existing literature (13). Recently, Du et al. reported that the risk of being LBW was increased approximately twofold in male newborns compared with female newborns after fresh ET (24). Another study by Raman et al. observed that pregnancy complications were strong positive predictors of birthweight (33). Therefore, considering that pregnancy complications possibly affect birthweight, we excluded women with complications from the study. In our study, there were significant differences between the EMT groups in a range of baseline characteristics, including maternal age, maternal BMI, infertility duration and others. However, these above-mentioned confounders had no impact on neonatal birthweight according to the regression model.

Notably, our present work has some strengths. Firstly, the maternal age in our study was limited to <35 years to eliminate the effect of advanced maternal age on adverse neonatal outcomes (34, 35). Secondly, excluding women with pregnancy complications makes the result more reliable and convincing. Third, based on the advantage of single center study, the potential bias caused by clinical protocols, EMT measurements, and laboratory operations could be minimized to a large extent. Last but not least, we adjusted for a number of potential confounders known to affect perinatal outcomes.

However, this investigation also has certain limitations. The main limitation of this study was its retrospective nature and did not explore the biological mechanism of EMT affecting the incidence of LBW. Another limitation was the lack of data on other risk factors for adverse neonatal outcomes, such as previous medication use, nutrition intake and lifestyle habit. In addition, although EMT was measured by the same team of trained sonographers providing routine sonographic evaluation of all our infertility patients, it is still a subjective measurement possibly affecting interpretation of the results.

## Conclusion

In conclusion, our study demonstrated that the birthweight and gestational age of neonates in EMT ≤ 7.5 mm group were significantly lower than those in EMT > 7.5-12 mm group and EMT >12 mm group. In addition, EMT was an independent risk factor for LBW in FET cycles, which may be caused by oxygen concentrations and spiral arterial vascular remodeling. Therefore, we suggest that women with a thin EMT after achieving pregnancy by IVF/ICSI-ET treatment should receive warrant more attention from obstetricians and pediatricians to reduce the risk of delivering a LBW newborns. Furthermore, a large prospective cohort studies with a longer follow-up period are needed to confirm our conclusion and explore the biological mechanism of EMT affecting the incidence of LBW.

## Data availability statement

The raw data supporting the conclusions of this article will be made available by the authors, without undue reservation.

## Ethics statement

The studies involving human participants were reviewed and approved by the Ethics Committee of the Northwest Women’s and Children’s Hospital. The patients/participants provided their written informed consent to participate in this study.

## Author contributions

JS contributed to the study design and critical revision of the manuscript. XX contributed to revision of the manuscript. TH analyzed the data and drafted the manuscript. ML, WL and PM contributed to acquisition of data. All authors contributed to the article and approved the submitted version.

## Acknowledgments

The authors thank the staff at the Center for Assisted Reproductive Technology of Northwest Women’s and Children’s Hospital for their support and cooperation.

## Conflict of interest

The authors declare that the research was conducted in the absence of any commercial or financial relationships that could be construed as a potential conflict of interest.

## Publisher’s note

All claims expressed in this article are solely those of the authors and do not necessarily represent those of their affiliated organizations, or those of the publisher, the editors and the reviewers. Any product that may be evaluated in this article, or claim that may be made by its manufacturer, is not guaranteed or endorsed by the publisher.
