# Corrigendum: Endometrial thickness is associated with low birthweight in frozen embryo transfer cycles: a retrospective cohort study of 8,235 singleton newborns

**DOI:** 10.3389/fendo.2022.1126063

**Published:** 2023-01-05

**Authors:** Tingting He, Mingzhao Li, Wei Li, Peng Meng, Xia Xue, Juanzi Shi

**Affiliations:** Assisted Reproduction Center, Northwest Women’s and Children’s Hospital, Xi’an, China

**Keywords:** Endometrial thickness, low birthweight, frozen embryo transfer, singleton, newborns

In the published article, there was an error the affiliation. Instead of “Assisted Reproduction Center, Northwest Women’s and Children’s Hospital Affiliated to Xi’an Jiaotong University, Xi’an, China”, it should be: “Assisted Reproduction Center, Northwest Women’s and Children’s Hospital, Xi’an, China”.

The authors apologize for this error and state that this does not change the scientific conclusions of the article in any way. The original article has been updated.

